# The Influence of the Use of Technological Waste and the Simulation of Material Lifetime on the Unnotched Impact Strength of Two Different Polymer Composites

**DOI:** 10.3390/ma15238516

**Published:** 2022-11-29

**Authors:** Jozef Dobránsky, Miroslav Gombár, Tomáš Stejskal

**Affiliations:** 1 Faculty of Manufacturing Technologies with a Seat in Presov, Technical University of Kosice, Sturova 31, 080 01 Presov, Slovakia; 2 Faculty of Management and Business, University of Presov, Namestie Legionarov 3, 080 01 Presov, Slovakia; 3 Faculty of Mechanical Engineering, Technical University of Kosice, Letna 9, 042 00 Kosice, Slovakia

**Keywords:** technological waste, simulation, material lifetime, impact strength, polymer composites

## Abstract

The article deals with the assessment of the impact of technological polymer waste resulting from plastic injection technology and the subsequent simulation of the lifetime of polymer products on the impact strength of two different polymer composites. Two different types of polymer composites used to produce plastic parts in the automotive industry were chosen for the research. Based on the evaluation of the PBT composite before exposing the specimens to elevated temperature, it can be concluded that the concentration of the recycled material in the virgin material only affects the values of the unnotched impact strength of the PBT composite when the recycled material accounts for at least 50 wt.%. The results of the unnotched impact strength of the test specimens exposed at 150 °C/500 h make it evident that the addition of recycled material to the virgin material significantly reduces the components’ service lives. The same trend could be observed with the second tested composite material, PPA.

## 1. Introduction

In their study, the authors Tomar and Maiti looked at the unnotched impact strength of a polymeric PBT blend containing up to 39% ABAS. By mixing the materials, the tensile properties were reduced, while the unnotched impact strength increased. Morphological studies showed good ABAS dispersion into the PBT matrix. The strength properties were dependent on PBT crystallinity and interphase adhesion between phases. The unnotched impact strength depended on the concentration of admixed rubber and the interparticle spacing of the discrete phase [1].

In his study, the author Szostak analyzed the effect of the method of PET and PBT mixture preparation. He found that the method of preparation had the greatest effect on the mechanical properties, especially the impact strength. Materials must first be mixed in the extrusion machine before they are processed in the injection machine. This improves the properties of the injected PET/PBT mixtures [2].

In their work, the authors Sharma et al. used two types of impact modifiers to monitor their effect on the unnotched impact strength of the modified materials. They compared ultra low density polyethylene grafted glycidyl methacrylate (ULDPE-g-GMA, IM1) and ethylene-methyl acrylate-glycidyl methacrylate (E-MA-GMA, IM2). The application of 2% ULDPE-g-GMA weight produced a better result compared to 2% E-MA-GMA weight in terms of unnotched impact strength. Therefore, 2% ULDPE-g-GMA weight is considered an optimized percentage for nanocomposite preparation. As an unnotched impact strength modifier, ULDPE-g-GMA reduces the yield strength and tensile modulus of pure PBT [3].

In their research, the authors Ambrosio et al. focused on the effect of the parameters of mixtures processed on a twin-screw extruder. In processing mixtures of polybutylene terephthalate acrylonitrile-butadiene-styrene with and without a reactive compatibilizer, they monitored the morphology and impact strength. In their study, they found that increasing the feed rate greatly reduced the ductile brittle transition temperature (DBTT) and slightly increased the impact strength at room temperature (RTIS) of compatible mixtures [4].

The main goal of the study by the authors Ajorloo et al. was to investigate the feasibility of using waste materials. They used different ratios of virgin and recycled PP. The authors found that increasing the concentration of recycled PP in the composite led to decreases in the mechanical properties, especially the ductility and impact strength. Due to the lower viscosity in the form of recycled PP, a better dispersion of the filler particles was observed by SEM analysis, leading to an improvement in tensile strength. In addition, the use of recycled material caused decreases in the percentage of crystallinity and the melting point. During the study, it was found that the use of a 20/80 to 40/60 ratio of recycled PP/virgin PP in composites adequately met the requirements of automotive parts, with advantages in environmental and economic aspects. Compared to composites filled with talc, the ductility of composites filled with fly ash is higher. The simultaneous use of talc and fly ash as a hybrid system results in higher ductility but lowers the impact strength compared to samples filled with talc. The use of recycled PP mixed with fly ash provides advantages in cost reduction and sustainable and ecological production [5].

Guo et al. observed the damage of polymer composites reinforced with wood fibers. They used waste WF and recycled HDPE (Re-HDPE) to understand the failure mechanism of wood-fiber-reinforced (WF) high-density polyethylene (HDPE) composites. Highly filled WF/Re-HDPE composites were prepared by extrusion. The results showed that acoustic emission (AE) technology can better understand the damage progression and fracture process of WF/Re-HDPE composites and determine the damage degree, damage accumulation, and damage mode. Matrix deformation, fiber breakage, interfacial delamination, fiber-to-matrix debonding, fiber pull-out, and matrix cracking were the dominant damage modes of highly filled WF/Re-HDPE composites under flexural loading, and the AE signal varied with different degrees of damage and modes of damage [6].

Plastics are cheap, easy to shape, and light. Plastics have substantially supplanted traditional materials in many areas. The problem of recycling is still a big challenge. There are both technological and economic challenges that hinder progress in this area. In their study, the authors Ignatyev et al. provided an overview of recycling, along with a future outlook using popular polymers such as polyolefins, poly(vinyl chloride), polyurethane, and poly(ethylene terephthalate). The different types of recycling, primary, secondary, tertiary, quaternary, and biological recycling, were discussed, together with related issues such as compatibility and cross-linking [7,8].

In addition to the positive effects on environmental sustainability, polymer recycling is also beneficial in terms of reducing production costs and material resources. The aim of the study by Jiun et al. was to find out how the recycling cycle affects the physical properties and mechanical properties of the thermoplastic polymer and thermoplastic elastomer polymer. The obtained results showed that the tensile strength and density decreased for the thermoplastic polymer, and the appearance changes were significant when the number of recycling cycles increased [9,10].

An article by Hugo et al. dealt with the formulation of composite materials for structural or semistructural applications using thermoplastic polymer waste. The mechanical and thermal properties of a proprietary blend of recycled polymers with a range of different fillers were investigated. The authors found that the addition of small amounts of mica to glass-fiber-reinforced composites showed significant synergy in the tensile strength and modulus [11,12].

The article was created based on a request from practice. The company that processes the composite materials in question wanted to verify their properties. At the same time, there is also an ecological side. Reusing waste material in the manufacturing process reduces the amount of waste in the automotive supply chain.

## 2. Experiment Preparation

For the analyses, two types of materials were selected from the group of polymer composites that are used for the production of moldings (connectors) intended for the automotive industry. The materials that form the matrices of the composites belong to the group of engineering (structural) or highly durable (high-tech) polymer composites. From the group of structural polymers, the PBT material (polybutylene terephthalate) and PPA material (polyphthalamide) were selected [13,14].

The first composite of polybutylene terephthalate (PBT), marketed as Ultradur^®^ B 4406 G6, consists of 30 wt.% glass fibers. It is designed for products requiring increased resistance to burning. An overview of the basic material properties of this material is given in Table 1.

The second composite, polyphthalamide (PPA), with the trade name ZYTEL^®^ HTN FR52G30NH NC010, is filled with 30% glass fibers and nonhalogenated additives: flame retardants, lubricants, and separation agents. Injection molds for the use of this type of material must be made of corrosion-resistant and wear-resistant steels. When processing the material, it is important to make sure that it is not in the injection unit for a long time because it can degrade. An overview of the basic material properties of the composite is given in Table 2.

Before the experimental measurement, it was necessary to prepare the test specimens, which consisted of mixing recycled material in the form of pulp with the virgin granulate. Mixing was carried out manually in specified ratios, corresponding to the recycled content in the virgin material, of 0, 10, 20, 30, 40, 50, 60, 80, and 100 wt.%. From the mixture (batches) prepared in this way, multipurpose type A test specimens were injected according to STS EN ISO 3167 or 1A specimens were injected according to STS EN ISO 527-2. The production of multipurpose test specimens in the form of double-sided blades and rectangular prisms for the Charpy unnotched impact strength test was carried out in accordance with STS EN ISO 294-1 [15].

The injection process took place on a standard Demag D 60–182 injection machine with a column structure. Both materials were dried before injection under the conditions indicated in their respective material data sheets. At the end of the production cycle, all specimens were conditioned under standard conditions of 23/50. To simulate the assessment of the lives of the moldings according to the recyclate content, half of the test specimens were exposed to an elevated temperature of 150 °C (PBT) or 230 °C (PPA) in a forced air circulation oven for 500 h prior to studying their performance.

**Figure 1:**
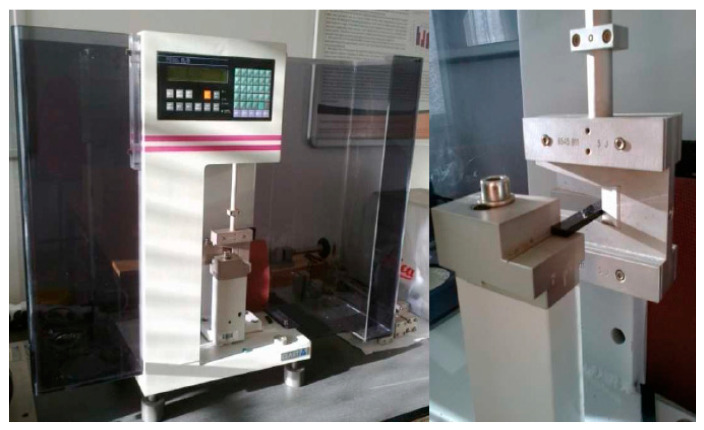
CEAST Resil 5.5 test device with sample location.

The determination of the unnotched impact strength by the Charpy method was carried out under the conditions specified in the STS EN ISO 179-1 standard. Before the test, the span of the instrument brackets was set, and the instrument was calibrated. Afterwards, a hammer was let down loosely to determine the friction losses. At the same time, it was checked whether the Ceast Resil 5.5 (Figure 1) instrument was able to perform the test at the prescribed impact velocity and whether it was in the correct range of the absorbed energy. For the purposes of the experimental measurement, we used an impact hammer with a nominal energy of 5 J for the PBT material before exposure to elevated temperature. A hammer with a nominal energy of 2 J was used for the PBT after exposure and the PPA before and after exposure [16,17,18].

Half of the test specimens intended to determine the unnotched impact strength at low temperatures were conditioned in the ProfiMaster PMU 0450 laboratory freezer at –30 °C for 24 h. A thorough type C puncture was recorded in all test specimens.

## 3. Evaluation of Results and Discussion

This chapter deals with the assessment of unnotched impact strength by the Charpy method. The unnotched impact strength was first measured at 23 °C on test specimens before and after exposure to elevated temperature. Subsequently, the bodies were exposed to a temperature of −30 °C for 24 h, and the unnotched impact strength at low temperatures was measured. The influence of the presence of the recycled material on the lives of the samples is also evaluated, where the lives were simulated by exposing the materials to an elevated temperature of 150 °C for 500 h (PBT) or 230 °C for 500 h (PPA), according to the internal regulations of the VW Group [19,20].

### 3.1. Evaluation of Unnotched Impact Strength by the Charpy Method for Composite PBT

**Figure 2:**
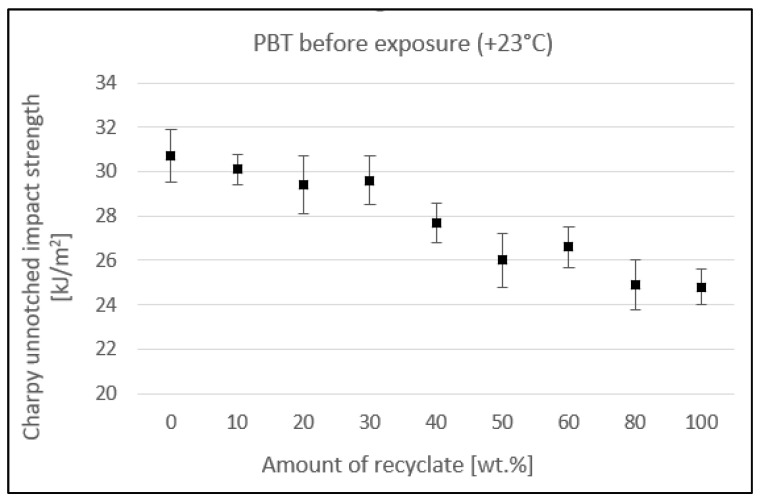
Dependence of the unnotched impact strength of PBT on the amount of recycled material before the samples were exposed to elevated temperature (+23 °C).

Figure 2 shows the impact of the amount of recyclate on the unnotched impact strength. As shown in the figure, the amount of recycled material in the virgin material affects the value of the unnotched impact strength. The unnotched impact strength value decreased from 30.7 kJ/m^2^ (virgin material) to 24.8 kJ/m^2^ when the recyclate accounted for 100% of the amount, representing an overall drop of 19.2%.

Up to 40 wt.% recycled material, the unnotched impact strength decreased slightly. With 50 wt.% recycled material, the value recorded a greater drop, falling from 30.7 kJ/m^2^ (virgin material) to 26 kJ/m^2^, a decrease of 15.3%.

**Figure 3:**
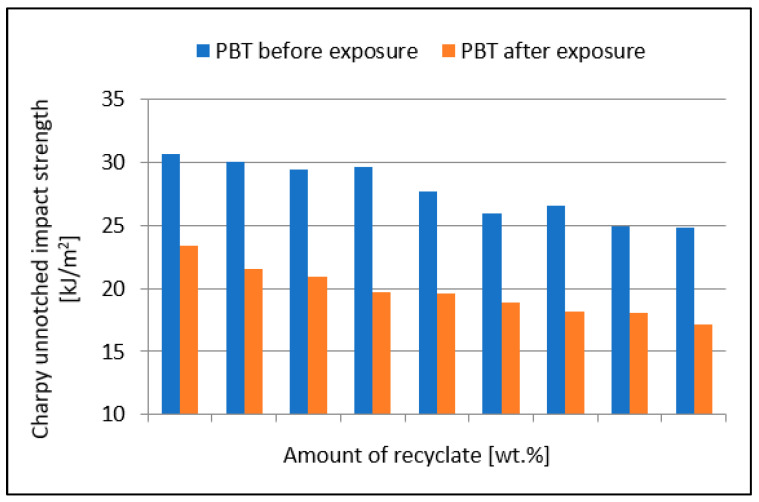
Dependence of the unnotched impact strength of PBT on the amount of recycled material before and after the samples were exposed to elevated temperature (+23 °C).

Figure 3 shows a comparison of the PBT composite unnotched impact strength values before and after exposure to elevated temperature. The value of unnotched impact strength after the specimens were exposed to elevated temperature decreased gradually from the value of 23.4 kJ/m^2^ (virgin material) to the value of 17.1 kJ/m^2^ (100% recyclate), which represented a reduction of 26.9%.

The value of the unnotched impact strength, shown in Figure 2, after the specimens were exposed to elevated temperature decreased, on average, by between 23.8 and 31% compared to unexposed specimens, which represented a significant reduction. The results also confirm the conclusions of Kim et al. [21].

**Figure 4:**
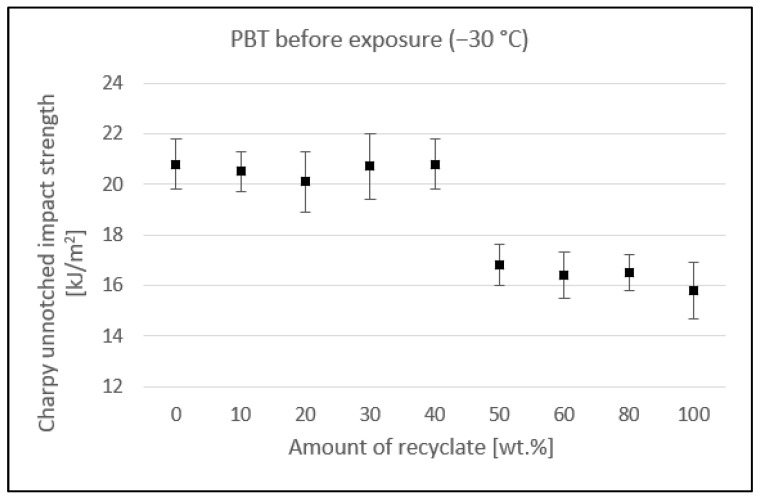
Dependence of the unnotched impact strength of PBT on the amount of recycled material before the samples were exposed to elevated temperature (−30 °C).

Figure 4 shows the effect of added recycled material on unnotched impact strength measured in the specimens exposed to −30 °C. Up to a concentration of 40 wt.% recycled material, the value of the unnotched impact strength fluctuated around 20.5 kJ/m^2^. At a 50 wt.% concentration of recycled material, it decreased to 16.8 kJ/m^2^, a drop of 19.2%. The further addition of recyclate caused only a marginal reduction to a final value of 15.8 kJ/m^2^, which represented an overall reduction of 24% compared to the virgin material.

**Figure 5:**
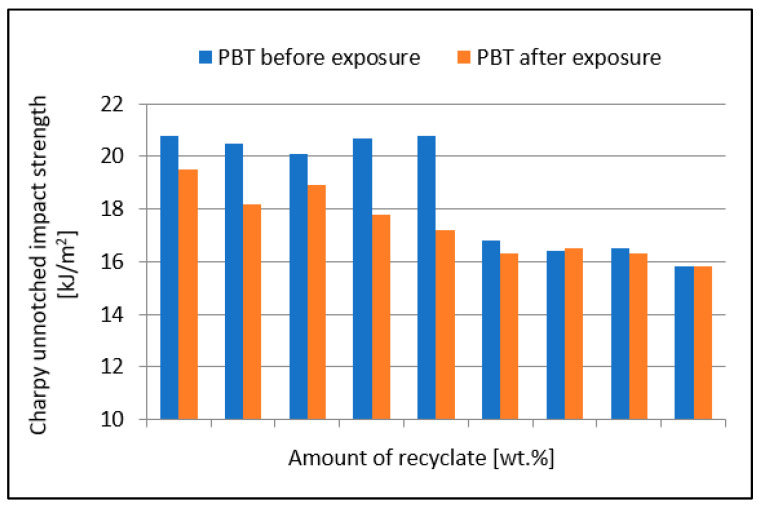
Dependence of the unnotched impact strength of PBT on the amount of the recycled material before and after the samples were exposed to elevated temperature (−30 °C).

Figure 5 shows a comparison of the unnotched impact strength values before and after exposure to elevated temperature, measured at the specimen temperature of −30 °C. The unnotched impact strength value was lower in specimens composed of up to 40 wt.% recycled material compared to specimens before exposure, where it decreased by 6.2% in the virgin material and by as much as 17.3% with the recyclate accounting for 40 wt.%. At concentrations between 50 and 100 wt.% of the recycled material, the unnotched impact strength value varied only marginally compared to the specimens before they were exposed to elevated temperature. The same finding was observed by Behalek et al. [22].

Based on the evaluation of the PBT composite before the specimens were exposed to elevated temperature, it can be concluded that the concentration of the recycled material in the virgin material only affects the values of the unnotched impact strength of the PBT composite when the recycled material accounts for at least 50 wt.%.

The results of the unnotched impact strength of the test specimens exposed to 150 °C/500 h make it evident that the addition of recycled material to the virgin material significantly reduces the components’ service lives.

### 3.2. Evaluation of Unnotched Impact Strength by the Charpy Method for Composite PPA

**Figure 6:**
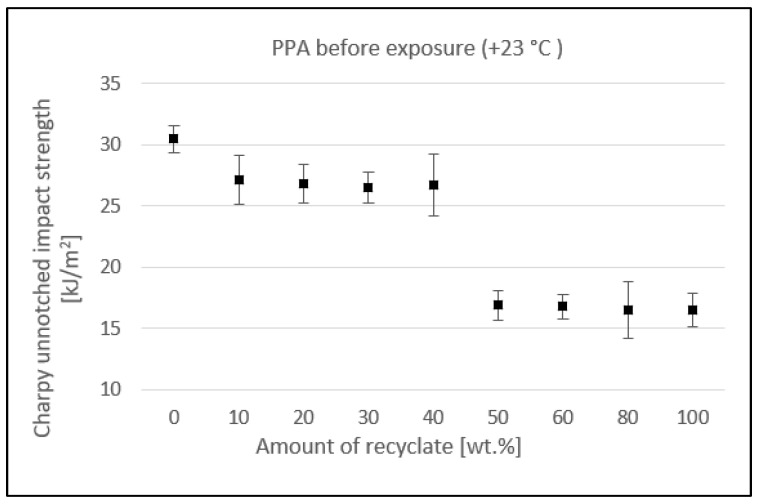
Dependence of the unnotched impact strength of PPA on the amount of recycled material before the samples were exposed to elevated temperature (+23 °C).

Figure 6 shows the effect of the recyclate concentration on the unnotched impact strength of the PPA composite. The virgin material value of unnotched impact strength decreased slightly up to a 40 wt.% concentration of the recycled material, from 30.5 kJ/m^2^ to 26.7 kJ/m^2^, a reduction of 12.5%. At a 50 wt.% concentration of the recycled material, the unnotched impact strength dropped rapidly to 16.9 kJ/m^2^, a reduction of 44.6% compared to virgin material. Adding further recycled material did not result in a further reduction in the unnotched impact strength.

**Figure 7:**
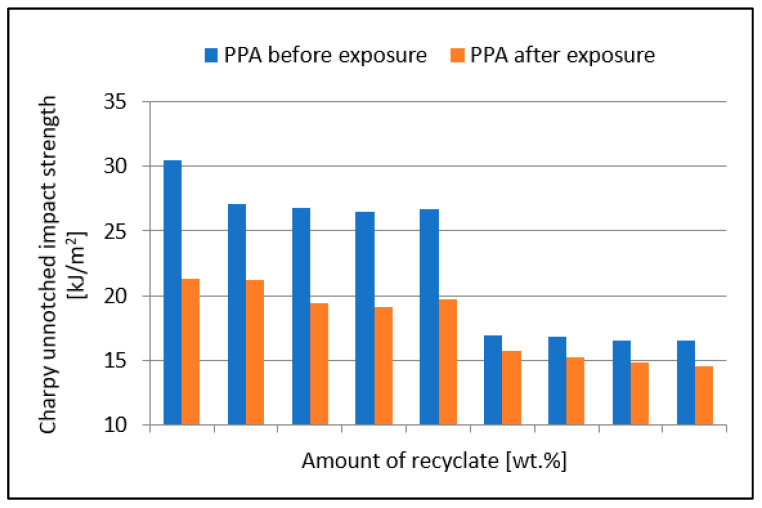
Dependence of the unnotched impact strength of PPA on the amount of recycled material before and after the samples were exposed to elevated temperature (+23 °C).

Figure 7 shows a comparison of the unnotched impact strength of the specimens before and after exposure to elevated temperature. The value of the unnotched impact strength of specimens exposed to elevated temperature in virgin material decreased marginally from the value of 21.3 kJ/m^2^ to 19.7 kJ/m^2^ at a 40 wt.% concentration of the recycled material, a reduction of 7.5%. At the recycled material concentration of 50 wt.%, the value dropped to 15.7 kJ/m^2^, a reduction of 26.3% compared to the virgin material. By adding more recycled material, the value of the unnotched impact strength decreased marginally.

The unnotched impact strength differed between specimens exposed to elevated temperature and those that were not yet exposed to elevated temperature up to the recycled material concentration of 40 wt.%. The difference in values ranged from 27.6 to 30.2%. At a recyclate concentration of 50 wt.% or more, no significant differences in unnotched impact strength values were recorded. The results also confirm the conclusions of Wang et al. [23] and Svetlik et al. [24].

**Figure 8:**
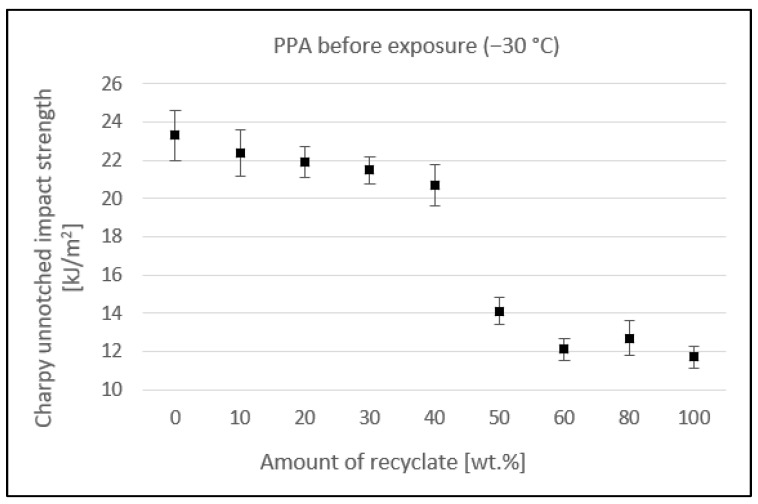
Dependence of the unnotched impact strength of PPA on the amount of recycled material before the samples were exposed to elevated temperature (−30 °C).

Figure 8 shows the effect of the addition of recycled material on the unnotched impact strength measured in specimens exposed to a temperature of −30 °C. The value of the unnotched impact strength up to 40 wt.% recycled material decreased from at least 23.3 kJ/m^2^ in virgin material to 20.7 kJ/m^2^, a reduction of 11.2%. At the recyclate concentration of 50 wt.%, it dropped to the value of 14.1 kJ/m^2^, a reduction of 39.5%. With the further addition of recyclate, it dropped only marginally, to a final value of 11.7 kJ/m^2^, which represented an overall reduction of 49.8% compared to the virgin material.

**Figure 9:**
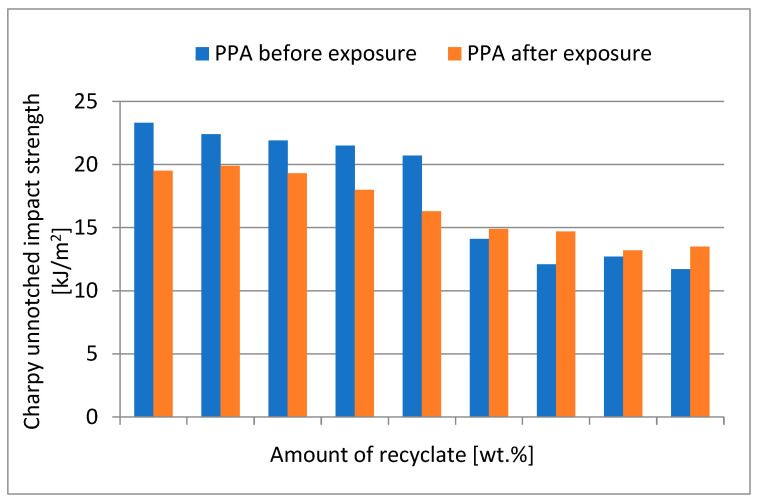
Dependence of the unnotched impact strength of PPA on the amount of recycled material before and after the samples were exposed to elevated temperature (−30 °C).

Figure 9 shows a comparison of the unnotched impact strength values before and after the exposure to elevated temperature, measured on specimens at a low temperature.

After the specimens were exposed to elevated temperature, they exhibited lower values of unnotched impact strength up to the recycled material concentration of 40 wt.% compared to the specimens before exposure, ranging from 11.9 to 21.3%. At a recyclate concentration of 50 wt.% or more, the unnotched impact strength of the specimens after exposure was slightly greater than it was before exposure. The results also confirm the conclusions of Khalili et al. [25] and Milosevic [26].

Based on the evaluation of the PPA composite before the specimens were exposed to elevated temperature, it can be concluded that the concentration of the recycled material in the virgin material only affects the values of the unnotched impact strength of the PPA composite when the recycled material accounts for at least 50 wt.%.

The unnotched impact strength results measured on the test specimens exposed to 230 °C/500 h make it evident that the addition of recycled material to the virgin material significantly reduces the components’ service lives. The results also confirm the conclusions of Hernandez-Diaz et al. [27].

The same trend could be observed in both cases involving specimens where measurements were performed at a low temperature. The values of the unnotched impact strength decreased in specimens both before and after exposure to elevated temperature.

## 4. Conclusions

The basic statistical analysis of the general model (1) that was used to predict the examined response y depending on the change in the studied independent variables, xi, in the nominal (state) and ordinal scale (the recycled material amount) was performed through an analysis of variance. The analysis of variance of the studied parameter, y, was a basic statistical analysis of the suitability of the general model that was used (1). An analysis of variance can, on one hand, analyze whether the variability due to random errors is significantly smaller than the variability in the measured values explained by the model. On the other hand, ANOVA results can be viewed in terms of the basic nature of ANOVA, testing the zero statistical hypothesis stating that neither of the effects used in the models (the state and the recycled material amount) had a significant effect on the change in the studied variable (y). The statistical analysis of the experimental results utilized a factorial analysis of variance, where the influence of the main effects of the independent variables and their mutual interaction were considered. The basic general ANOVA table is shown in Table 3.

(1) $$\overset{⌢}{y}=b_{0}\cdot x_{0}+\sum_{j=1}^{N}b_{j}\cdot x_{j}+\sum_{\begin{array}{l} u,j=1\\ u\neq j \end{array}}^{N}b_{uj}\cdot x_{u}\cdot x_{j}$$

**Figure 10:**
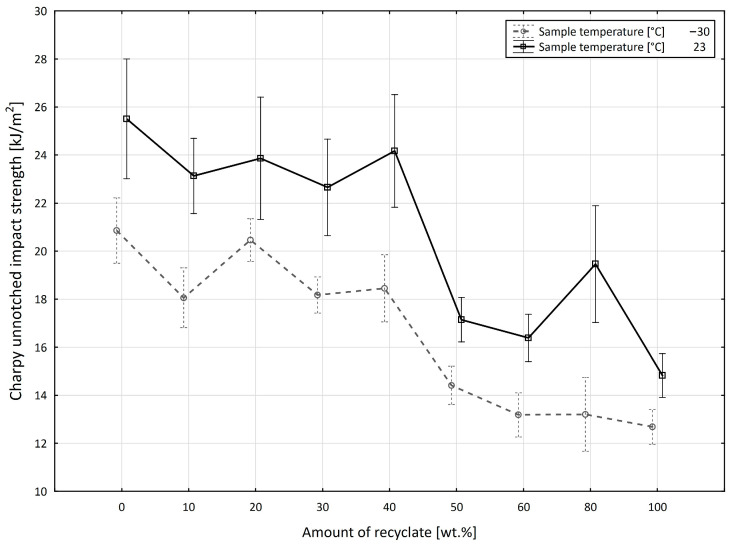
Dependence of the mutual interaction of the amount of recyclate and the temperature of the sample on the value of the unnotched impact strength of PPA.

In order to better understand the interaction of the independent input variables on the studied value of the unnotched impact strength expressed by the a_cU_ value (kJ·m^−2^) in two independent test procedures, we analyzed both tests conducted for PPA and PBT separately. In the first unnotched impact strength test performed on the PPA material, based on the applied analytical method (ANOVA), we concluded that the considered independent input variables (the recycled material amount, the specimen temperature, and the exposure) at the selected level of significance (α = 0.05) significantly affected the conditioned value of the unnotched impact strength. In the sense of the general model (1), where the effect of the mutual interactions of the input factors was also considered, the mutual interaction of the amount of recycled material and the temperature of the specimen (*p* = 0.000) contributed 1.720% of the change in the conditional value of the studied variable. Another significant interaction defined by the mutual relationship between the amount of recycled material and the exposure (*p* = 0.000) accounted for 6.880% of the change in the conditional value of the studied variable. The interaction of the specimen temperature and the exposure (*p* = 0.000) accounted for 1.130% of the change in the conditional value of the studied variable, and finally the interaction of all three considered input variables (*p* = 0.001) accounted for 1.130% of the change in the conditional value of the studied variable. At the same time, it has to be said that the quantity of the recycled material, accounting for 41.950% of the change in the conditional value of the studied variable, had the greatest impact on the change in the value of the energy of the unnotched impact strength in the PPA test. For the sake of completeness, the model error accounted for 13.170% of the change, i.e., the model (1) defined for the experimentally obtained values can explain 86.830% of the variability in the studied a_cU_ (kJ·m^−2^) variable. In view of the fact that the previous analysis confirmed the statistical significance of the interactions, it is necessary and needed to also consider the simultaneous effects of the independent input variables. As documented in Figure 10, there were significant differences between the specimen temperatures of −30 °C and +23 °C containing individual quantities of recycled material (from 0 to 100%). First, it can be observed that the average value of the unnotched impact strength for the specimen temperature of +23 °C (20.178 ± 0.802 kJ·m^−2^) was higher than the average value of the unnotched impact strength at the specimen temperature of −30 °C (16.610 ± 0.556 kJ·m^−2^) over the entire range of the amount of recyclate that was used. This average deviation was 4.185 kJ·m^−2^. However, under the effect of the amount of recycled material used, we can see that the difference in the value of the unnotched impact strength between the specimen temperature was the smallest for +23 °C and −30 °C at a 100 wt.% amount of recycled material, and this difference was 2.143 ± 0.821 kJ·m^−2^ (*p* = 0.001). On the contrary, the highest value of the studied difference was observed when the recycled material accounted for 80 wt.%, with a value of 6.259 ± 1.984 kJ·m^−2^ (*p* = 0.000). It should be mentioned that all differences in the values of the unnotched impact strength were significant at the selected level of significance (α = 0.05).

**Figure 11:**
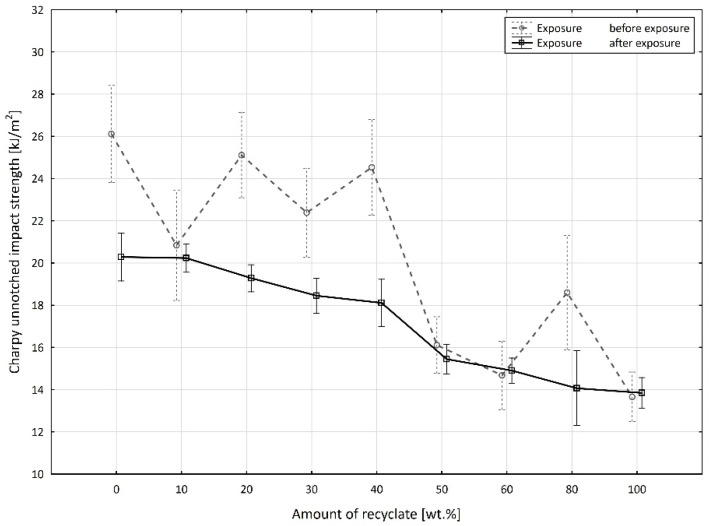
Dependence of the mutual interaction of the amount of recyclate and the exposure of the sample on the value of the unnotched impact strength of PPA.

When examining the mutual interaction between the amount of recycled material and the exposure (Figure 11), it can be said that, with the exception of the recycled material accounting for 60 wt.% and 100 wt.%, the value of the unnotched impact strength was higher in the state of the specimen before exposure. The average value of the difference within the entire range of the recycled material amount was 3.046 kJ·m^−2^. Just as in the previous case, it was also important to monitor the amount of recycled material. The maximum difference in the values of the studied variable was achieved at 40 wt.%, with this difference being significant at the selected level of significance (*p* = 0.000) and reaching the value of 6.430 ± 1.697 kJ·m^−2^. The next values of the a_cU_ variable difference were found when the recycled material accounted for 20 wt.% (5.833 ± 1.331 kJ·m^−2^, *p* = 0.000) and when the recycled amount was 0 wt.%, with a value of 5.826 ± 1.719 kJ·m^−2^ (*p* = 0.000). On the other hand, two negative differences in the unnotched impact strength value, namely with the amount of the recyclate accounting for 60 wt.% (−0.231 ± 1.108 kJ·m^−2^, *p* = 0.708) and 100 wt.% (−0.188 ± 0.945 kJ·m^−2^, *p* = 0.761) were statistically insignificant. We observed other statistically insignificant differences in the evaluated unnotched impact strength at 10 wt.% (0.610 ± 1.646 kJ·m^−2^, *p* = 0.166) and 50 wt.% (0.666 ± 1.026 kJ·m^−2^, *p* = 0.281).

**Figure 12:**
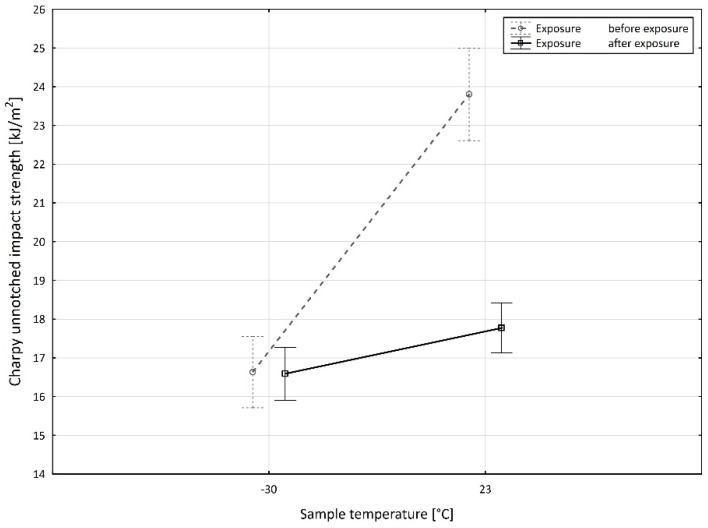
Dependence of the mutual interaction of the sample temperature and the exposure of the sample on the value of the unnotched impact strength of PPA.

When examining the mutual interaction of the specimen temperature and exposure (Figure 12), without considering the effect of the recycled material amount, we can see that at the specimen temperature of −30 °C there was no significant difference in the difference between the studied value of the unnotched impact strength, a_cU_ (0.045 ± 0.799 kJ·m^−2^, *p* = 0.878).

**Figure 13:**
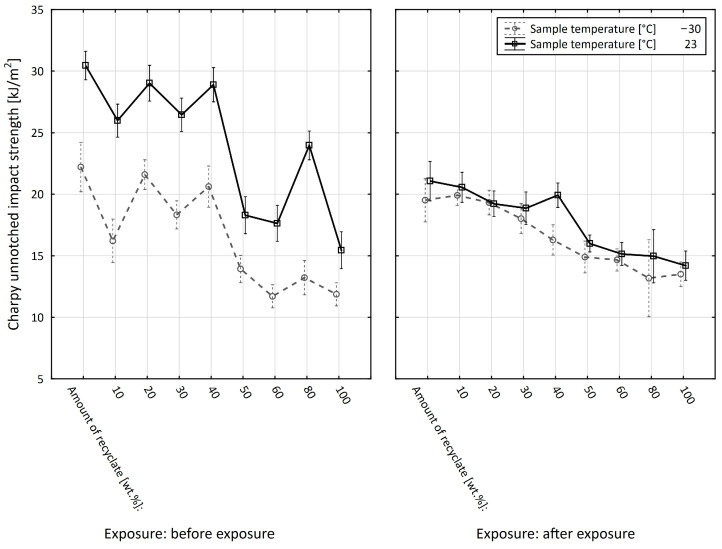
Dependence of the mutual interaction of the sample temperature and the exposure of the sample on the value of the unnotched impact strength of PPA.

Furthermore, Figure 13 shows that at the specimen temperature of +23 °C the value of the unnotched impact strength before exposure (23.802 ± 1.195 kJ·m^−2^) was significantly higher than the value of the unnotched impact strength after exposure (17.771 ± 0.644 kJ·m^−2^). This difference was also significant (*p* = 0.000) at the selected level of significance of *p* = 0.05.

When analyzing all three input variables at the same time, as the last significant interaction (Figure 13), we observed the highest value of the difference in the studied value of unnotched impact strength with the recyclate accounting for 20 wt.%, a specimen temperature of +23 °C, and between the states before and after the exposure. This difference reached 9.784 ± 0.644 kJ·m^−2^ (*p* = 0.000). The second most significant difference, with a value of 9.393 ± 1.384 kJ·m^−2^ (*p* = 0.000) was between the states before and after the exposure of the specimens with 0 wt.% consisting of the recyclate and a temperature of +23 °C. Significant values for the differences in the unnotched impact strength could be observed between the states before and after the exposure in the specimens with the recyclate accounting for 80 wt.% with the specimen temperature of +23 °C (9.005 ± 1.668 kJ·m^−2^, *p* = 0.000) and with the recyclate accounting for 40 wt.% with the specimen temperature of +23 °C (8.975 ± 1.188 kJ·m^−2^, *p* = 0.000). For all the above relationships with a specimen temperature of +23 °C, the unnotched impact strength values were higher in the state before the exposure. The opposite trend, i.e., greater values of unnotched impact strength in the state after the exposure compared to the state before the exposure, was observed only under the specimen temperature of −30 °C, namely with the recyclate accounting for 10 wt.% (3.688 ± 1.284 kJ·m^−2^, *p* = 0.000), with the recyclate accounting for 60 wt.% (2.953 ± 0.911 kJ·m^−2^, *p* = 0.001), and with the recyclate accounting for 100 wt.% (1.618 ± 0.966 kJ·m^−2^, *p* = 0.044).

The initial analysis of the PBT unnotched impact strength test indicated that the change in the studied variable, a_cU_ (kJ·m^−2^), was significantly influenced by the amount of recycled material (*p* = 0.000), with a 9.650% effect in model (1); specimen temperature (*p* = 0.000), with a 23.450% effect, as well as the exposure (*p* = 0.000), with a 22.720% effect. Compared to the previous analysis of PPA, the most striking difference was the reduced effect of the recyclate amount. When we considered the interactions of mutually independent input variables within the studied model (1), the change in the value of the unnotched impact strength was significantly influenced by the interaction between the amount of recycled material and the exposure (*p* = 0.005), with a 2.260% effect; the interaction of the specimen temperature and the exposure (*p* = 0.000), with a 6.560% effect; and the interaction of the recycled material amount, the specimen temperature, and the exposure (*p* = 0.018), with a 1.870% effect. In contrast to PPA, in the case of PBT, no significant effect resulted from the interaction between the amount of recycled material and the temperature of the specimen (*p* = 0.183), and at the same time there was a higher share of model error (32.350%) in PBT than in the PPA models. When we considered the basic input variables as separate effects, the average value of the unnotched impact strength energy for all specimens was 21.198 ± 0.568 kJ·m^−2^. The highest value of unnotched impact strength was achieved by a specimen with 20 wt.% recycled material, namely 23.877 ± 1.651 kJ·m^−2^, and the lowest value (18.839 ± 1.506 kJ·m^−2^) was achieved by specimens with 60 wt.% recycled material. However, the unnotched impact strength values were relatively homogeneous in terms of the amount of recycled material, and the difference between the minimum and the maximum was 26.743%. In terms of the specimen temperature as a separate effect at −30 °C, the unnotched impact strength value reached 18.546 ± 0.511 kJ·m^−2^, and at +23 °C the values reached 23.849 ± 0.859 kJ·m^−2^. The difference, with a value of 5.304 ± 0.685 kJ·m^−2^, was significant at the level of significance of ± = 0.05 (*p* = 0.000). In terms of the exposure, as the last main effect that was studied, we achieved higher values of unnotched impact strength (23.808 ± 0.852 kJ·m^−2^) in specimens in the state before the exposure in contrast to specimens after the exposure (18.588 ± 0.532 kJ·m^−2^), while the difference was significant at the selected level of significance (*p* = 0.000). The first significant interaction between the amount of recycled material and the exposure points to the fact that higher unnotched impact strength values were always achieved in specimens prior to the exposure. Differences between the states before and after the exposure were highest when the recyclate accounted for 10 wt.% (7.629 ± 1.983 kJ·m^−2^), followed by the difference at 0 wt.% recyclate (6.845 ± 2.034 kJ·m^−2^) and at 50 wt.% recyclate (6.720 ± 1.976 kJ·m^−2^). All these differences were significant at the chosen level of significance. On the other hand, the minimum difference in the unnotched impact strength value between the states before and after the exposure was achieved when the recyclate accounted for 80 wt.% (2.025 ± 2.017 kJ·m^−2^). However, this difference was not statistically significant (*p* = 0.052). The second smallest difference was the difference when the recyclate accounted for 20 wt.%, with a value of 3.694 ± 2.249 kJ·m^−2^ (*p* = 0.000), followed by the difference when the recyclate accounted for 30 wt.%, with a value of 4.469 ± 2.328 kJ·m^−2^ (*p* = 0.000). The second significant interaction, namely between the exposure and the specimen temperature indicates that specimens before the exposure achieved higher mean unnotched impact strength values than after the exposure. Specifically, for a specimen temperature of −30 °C, this difference was 2.416 ± 0.678 kJ·m^−2^ (*p* = 0.000), and for a specimen temperature of +23 °C this difference was 8.024 ± 0.886 kJ·m^−2^ (*p* = 0.000).

**Figure 14:**
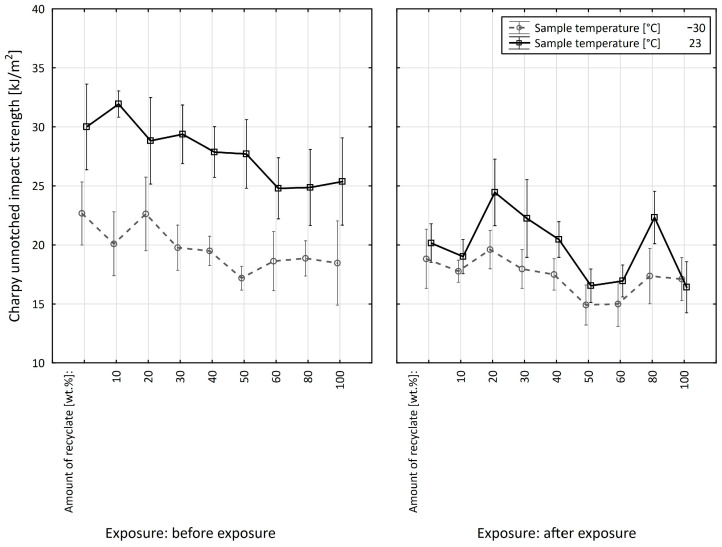
Dependence of the mutual interaction of the sample temperature and the exposure of the sample on the value of the unnotched impact strength of PBT.

In the mutual interaction of all input variables in PBT, as in the previous cases, the unnotched impact strength values for specimens before the exposure were higher compared to specimens after the exposure. In terms of their size, we observed the maximum difference in unnotched impact strength at 10 wt.% of abrasive with a specimen temperature of +23 °C, with a value of 12.930 ± 1.283 kJ·m^−2^ (*p* = 0.000). In order, the second highest value relating to the unnotched impact strength was achieved when the recyclate accounted for 50 wt.% with a temperature of +23 °C (11.170 ± 2.163 kJ·m^−2^; *p* = 0.000), followed by the difference in the unnotched impact strength values at 0 wt.% of the abrasive and, as in previous cases, the specimen temperature of +23 °C (9.848 ± 2.628 kJ·m^−2^; *p* = 0.000). On the contrary, the smallest values of the differences in the unnotched impact strength of specimens in the states before and after the exposure were achieved when the recyclate accounted for 100 wt.% with a specimen temperature of −30 °C (1.358 ± 2.935 kJ·m^−2^; *p* = 0.356), when the recyclate accounted for 80 wt.% with a specimen temperature of −30 °C (1.500 ± 1.917 kJ·m^−2^; *p* = 0.308), when the recyclate accounted for 30 wt.% with a specimen temperature of −30 °C (1.803 ± 1.779 kJ·m^−2^; *p* = 0.220), and when the recyclate accounted for 40 wt.% with a specimen temperature of −30 °C (1.993 ± 1.286 kJ·m^−2^; *p* = 0.176), all of which, however, were statistically insignificant. Interestingly, the differences in the value of the unnotched impact strength at −30 °C reached an average value of 2.456 kJ·m^−2^, while for the specimen temperature of +23 °C the average value of the unnotched impact strength difference was 8.024 kJ·m^−2^, from which it was possible to draw one of the partial conclusions that at the specimen temperature of −30 °C neither the exposure nor the amount of recycled material had a significant effect on the change in the value of the unnotched impact strength. A graphical representation of the complex effect of the input variables (the recycled material amount, the specimen temperature, and the exposure) on the change in the value of the unnotched impact strength is given in Figure 14.

**Figure 15:**
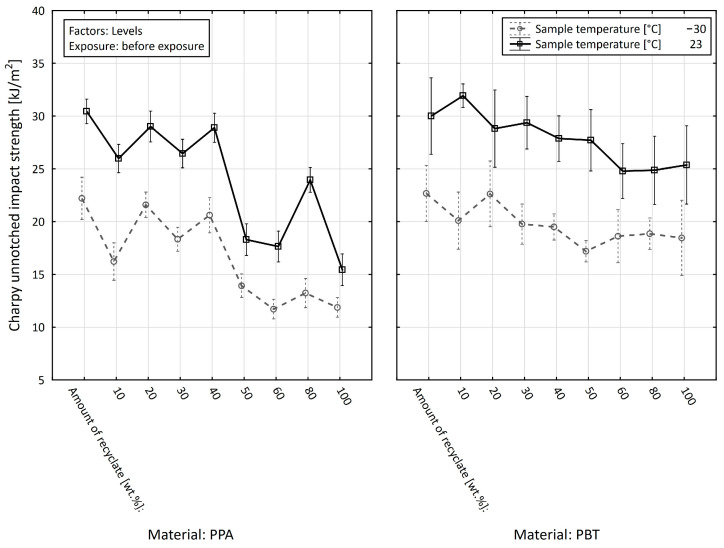
Effect of the materials on the impact test value of the unnotched impact strength, depending on the amount of recycled material and the specimen temperature before the exposure.

**Figure 16:**
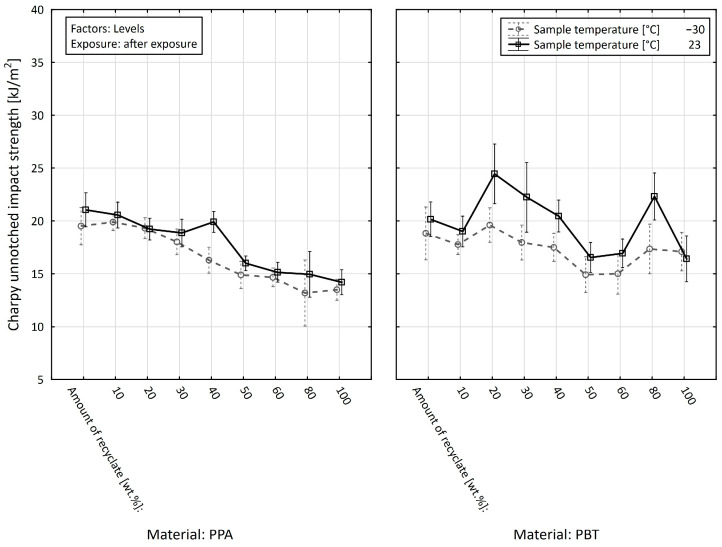
Effect of the materials on the impact test value of the unnotched impact strength depending on the amount of the recycled material and the specimen temperature after the exposure.

Finally, we analyzed the effect of the material (PPA or PBT) on the impact test value of the unnotched impact strength, a_cU_ (kJ·m^−2^), depending on the amount of recycled material, the specimen temperature, and the exposure, with the exposure being the dividing factor (Figure 15 and Figure 16).

If, in the first step, we focused on the value of the parameter studied in the specimens before the exposure, we arrived at the conclusion that the average value of the impact test of the unnotched impact strength (regardless of the specimen temperature and the amount of recycled material) in the case of PPA reached the value of 20.136 ± 0.915 kJ·m^−2^ and in the case of PBT reached the value of 23.808 ± 0.852 kJ·m^−2^. In the unnotched impact strength test in specimens after the exposure, the mean value in the case of PPA was 17.179 ± 0.472 kJ·m^−2^, and in the case of PBT it was 18.588 ± 0.532 kJ·m^−2^. If we also started to consider the effect of the amount of the recycled material and the specimen temperature, the average difference between PPA and PBT in the specimens before the exposure reached −1.409 kJ·m^−2^, which means that on average we achieved higher values of the studied a_cU_ (kJ·m^−2^) variable in PBT.

On closer analysis, we arrived at the conclusion that there were statistically insignificant differences at the chosen level of significance (α = 0.05) between the PPA and PBT specimens before the exposure at −30 °C (0.065 ± 1.435 kJ·m^−2^) when the recyclate accounted for 30 wt.%, at −30 °C (−0.015 ± 1.492 kJ·m^−2^) when the recyclate accounted for 50 wt.%, at −30 °C (−0.333 ± 1.403 kJ·m^−2^) when the recyclate accounted for 60 wt.%, and at −30 °C (−0.287 ± 1.317 kJ·m^−2^) when the recyclate accounted for 20 wt.%. On the other hand, the maximum difference in the value of the unnotched impact strength between PPA and PBT before the exposure was found when the recyclate accounted for 80 wt.% and the specimen temperature was +23 °C (−7.360 ± 2.191 kJ·m^−2^), followed by the difference in specimens with the recyclate accounting for 20 wt.% at +23 °C (−5.217 ± 1.925 kJ·m^−2^). In addition to the negative differences, there were situations in which the unnotched impact strength values of the PPA materials were higher than those of the PBT materials, both in specimens in which the recyclate accounted for 10 wt.% and, at the same time, with specimen temperatures of −30 °C (2.131 ± 0.867 kJ·m^−2^) and +23 °C (1.553 ± 1.332 kJ·m^−2^).

When, in the same way, we analyzed the value of the notch toughness, a_cU_ (kJ·m^−2^), between the PPA and PBT materials in the state after the exposure, we arrived at the conclusion that the difference between the studied materials (PPA and PBT) reached an average value of −3.484 kJ·m^−2^, which led us to a similar conclusion that the notch toughness value of PBT is on average higher than that of the PPA materials. However, in the state after the exposure, we found significantly higher differences than in the previous case. The maximum value of the difference in unnotched impact strength (−9.925 ± 2.604 kJ·m^−2^) was recorded when the recyclate accounted for 100 wt.% and the specimen temperature was +23 °C. In terms of the sizes of the differences, the second most significant, when the recyclate accounted for 50 wt.% and the specimen temperature was +23 °C (−9.420 ± 2.203 kJ·m^−2^), was followed by the difference when the recyclate accounted for 60 wt.% and the specimen temperature was +23 °C (−7.160 ± 2.030 kJ·m^−2^), the difference when the recyclate accounted for 60 wt.% and the specimen temperature was −30 °C (−6.915 ± 1.721 kJ·m^−2^), and the difference when the recyclate accounted for 100 wt.% and the specimen temperature was −30 °C (−6.588 ± 2.254 kJ·m^−2^). It was also interesting that at lower levels of the recycled material, in most cases, the differences were statistically insignificant. When the recyclate accounted for 0 wt.%, the difference value with a specimen temperature of −30 °C was −0.465 ± 2.335 kJ·m^−2^, and with a specimen temperature of +23 °C it was 0.445 ± 2.394 kJ·m^−2^. When the recyclate accounted for 20 wt.% and the specimen temperature was −30 °C, it was −1.034 ± 2.159 kJ·m^−2^, and with a specimen temperature of +23 °C, it was 0.199 ± 2.564 kJ·m^−2^. When the recyclate accounted for 30 wt.% and the specimen temperature was −30 °C, it was −1.443 ± 1.522 kJ·m^−2^, and when the specimen temperature was +23 °C, it was −2.928 ± 1.923 kJ·m^−2^. The last value of the recycled material amount, with statistically insignificant differences in the values of unnotched impact strength at both specimen temperatures, was when the recyclate accounted for 40 wt.%. For the specimen temperature of −30 °C, it was specifically 1.120 ± 1.456 kJ·m^−2^, and for the specimen temperature of +23 °C, it was 1.024 ± 1.769 kJ·m^−2^. With further increases in the amount of recycled material, the differences were already significant at the selected level of significance (α = 0.05).

## Figures and Tables

**Table 1:** Typical properties of ULTRADUR^®^ B 4406 G6.

Properties	Value	Unit
Density	1650	kg/m^3^
Melt volume-flow rate (MVR) (275 °C; 2.16 kg)	8	cm^3^/10 min
Tensile modulus	11,300	MPa
Tensile stress at break	145	MPa
Tensile strain at break	2.3	%
Charpy unnotched impact strength (+23 °C)	30	kJ/m^2^
Charpy unnotched impact strength (−30 °C)	25	kJ/m^2^

**Table 2:** Typical properties of ZYTEL^®^ HTN FR52G30NH NC010.

Properties	Value	Unit
Density	1.44	g/cm^3^
Tensile modulus	10,500	MPa
Tensile stress at break	150	MPa
Tensile strain at break	2.2	%
Charpy unnotched impact strength (+23 °C)	45	kJ/m^2^
Charpy unnotched impact strength (−30 °C)	40	kJ/m^2^

**Table 3:** Basic analysis of an ANOVA.

Source	DF	Sum of Squares	Mean Square	F Ratio	Prob > F
Model	DF_Model_ = a − 1	S_Model_	MS_Model_ = S_Model_/DF_Model_	F = MS_Model_/MS_Error_	p_M_
Error	DF_Error_ = N − a	S_Error_	MS_Error_ = S_Error_/DF_Error_		
C. Total	DF_C.Total_ = N − 1	S_C.Total_	MS_C.Total_ = S_C.Total_/DF_C.Total_		

## Data Availability

Not applicable.
